# Detection of *BRCA1* and *BRCA2* germline mutations in Japanese population using next-generation sequencing

**DOI:** 10.1002/mgg3.120

**Published:** 2014-12-04

**Authors:** Yosuke Hirotsu, Hiroshi Nakagomi, Ikuko Sakamoto, Kenji Amemiya, Hitoshi Mochizuki, Masao Omata

**Affiliations:** 1 Genome Analysis Center, Yamanashi Prefectural Central HospitalKofu, Japan; 2 Department of Breast Surgery, Yamanashi Prefectural Central HospitalKofu, Japan; 3 Department of Gynecology, Yamanashi Prefectural Central HospitalKofu, Japan; 4 Pathology Division, Laboratory Department, Yamanashi Prefectural Central HospitalKofu, Japan; 5 Department of Gastroenterology, Yamanashi Prefectural Central HospitalKofu, Japan; 6 Graduate School of Medicine, University of TokyoTokyo, Japan

**Keywords:** *BRCA1*, *BRCA2*, diagnostic, familial, Japanese, next-generation sequencing

## Abstract

Tumor suppressor genes *BRCA1* and *BRCA2* are the two main breast and ovarian cancer susceptibility genes, and their genetic testing has been used to evaluate the risk of hereditary breast and ovarian cancer (HBOC). While several studies have reported the prevalence of *BRCA1* and *BRCA2* mutations in Japanese populations, there is insufficient information about deleterious mutations compared with western countries. Moreover, because many rare variants are found in *BRCA1* and *BRCA2*, both of which encode large proteins, it is difficult to sequence all coding regions using the Sanger method for mutation detection. In this study, therefore, we performed next-generation sequencing (NGS) analysis of the entire coding regions of *BRCA1* and *BRCA2* in 135 breast and/or ovarian cancer patients. Deleterious *BRCA1* and *BRCA2* mutations were detected in 10 patients (7.4%) by NGS analysis. Of these, one mutation in *BRCA1* and two in *BRCA2* had not been reported previously. Furthermore, a *BRCA2* mutation found in a proband was also identified in two unaffected relatives. These data suggest the utility of screening *BRCA1* and *BRCA2* mutations by NGS in clinical diagnosis.

## Introduction

Genetic testing for hereditary breast and ovarian cancer (HBOC) susceptibility has clinical importance for cancer prevention. Indeed, approximately 5–10% of breast and ovarian cancer patients are likely to be hereditary (Cancer Genome Atlas Research Network 2011; Cancer Genome Atlas Network 2012). Previous studies showed that *BRCA1* (MIM #113705) and *BRCA2* (MIM #600185) are the two major breast and ovarian cancer susceptibility genes (Easton et al. 1993; Narod et al. 1995; King et al. 2003), accounting for approximately 15% of inherited cases (Couch et al. 2014; Kanchi et al. 2014). Additionally, inherited mutations in *TP53*,* PTEN*,* STK11*, and *CDH1* are associated with moderately high risks of breast cancer in the context of Li-Fraumeni syndrome, Cowden syndrome, Peutz–Jeughers syndrome, and hereditary diffuse gastric cancer syndrome, respectively (FitzGerald et al. 1998; Hearle et al. 2006; Schrader et al. 2008; Gonzalez et al. 2009; Walsh et al. 2010). Germline mutations in DNA repair genes such as *PALB2*,* ATM*, and *CHEK2* also confer a risk for breast and ovarian cancer (Couch et al. 2014; Kanchi et al. 2014).

Genetic linkage studies localized *BRCA1* and *BRCA2* to chromosomes 17q and 13q, respectively (Hall et al. 1990; Wooster et al. 1994), and the genes were subsequently cloned (Miki et al. 1994; Tavtigian et al. 1996) and shown to play a role in DNA damage repair and the regulation of genomic stability. The functional importance of BRCA1 and BRCA2 in vivo is supported by mouse models. For instance, *Brca1* or *Brca2* homozygous null mutants are embryonic lethal with growth retardation (Hakem et al. 1996; Connor et al. 1997; Suzuki et al. 1997), while *Brca1* and *Brca2* conditional knockout mice models showed that loss of Brca1 and Brca2 enhances tumorigenesis. This indicates that *BRCA1* and *BRCA2* are tumor suppressor genes (Xu et al. 1999; Jonkers et al. 2001).

BRCA1 (1863 amino acids) and BRCA2 (3418 amino acids) are large proteins with many repetitive elements (Welcsh and King 2001). More than 1800 distinct mutations have been reported in *BRCA1* and over 2,000 in *BRCA2* in the Breast Cancer Information Core (BIC) database (Couch et al. 2014). A mutation was regarded to be deleterious if it led to premature truncation and loss of normal protein function. Because *BRCA1* and *BRCA2* loss of function mutations scattered throughout the genes, it is necessary to screen all coding regions of both genes in a genetic diagnosis of HBOC. However, this is an expensive and time-consuming process using the Sanger method. Furthermore, genetic testing of *BRCA1* and *BRCA2* has not been extensively performed in Japan compared with western countries, partly because genetic testing costs are currently no longer covered by health insurance. The present study therefore used next-generation sequencing (NGS) technology to determine the prevalence of breast and ovarian cancer patients carrying germline mutations in *BRCA1* and *BRCA2*.

## Materials and Methods

### Patients and sample preparation

Peripheral blood samples were obtained from 135 breast and/or ovarian cancer patients and two unaffected individuals who attended Yamanashi Prefectural Central Hospital (Yamanashi, Japan) between 2013 and 2014. Lymphocytes were isolated following centrifugation of blood samples at 820*g* at 25°C for 10 min. Peripheral blood lymphocytes were stored at −80°C until required for DNA extraction. Total DNA was extracted from lymphocytes using the QIAamp® DNA Blood Mini kit (Qiagen, Tokyo, Japan) or QIAamp® DNA Blood Mini QIAcube Kit (QIAGEN) with the QIAcube (QIAGEN). The concentration of DNA was determined using the Nano Drop 2000 spectrophotometer (Thermo Fisher Scientific, Yokohama, Japan). Informed consent was obtained from all subjects, and this study was approved by the institutional review board at Yamanashi Prefectural Central Hospital.

### Targeted next-generation sequencing

For targeted NGS analysis, the Ion AmpliSeq™ *BRCA1* and *BRCA2* Panel (Life Technologies, Tokyo, Japan) containing 167 primer pairs in three pools was used. Multiplex PCR was performed using 50–100 ng genomic DNA with a premixed primer pool and Ion AmpliSeq™ HiFi master mix (Ion AmpliSeq™ Library Kit 2.0) for 2 min at 99°C, followed by 19 cycles of 99°C for 15 sec and 60°C for 4 min, ending with a holding period at 10°C. The PCR amplicons were treated with 2 *μ*L FuPa reagent to partially digest primer sequences and phosphorylate the amplicons at 50°C for 10 min, followed by 55°C for 10 min, then 60°C for 20 min. The amplicons were ligated to adapters with the diluted barcodes of the Ion Xpress™ Barcode Adapters kit (Life Technologies) for 30 min at 22°C then 72°C for 20 min. Adaptor ligated amplicon libraries were purified using Agencourt® AMPure® XP reagents (Beckman Coulter, Tokyo, Japan). The library concentration was determined using an Ion Library Quantitation Kit (Life Technologies), then each library was diluted to 8–16 pmol/L and the same amount of libraries was pooled for one sequence reaction. Next, emulsion PCR was carried out using the Ion OneTouch™ System and Ion OneTouch™ 200 Template Kit v2 (Life Technologies) according to the manufacturer's instructions. Template-positive Ion Sphere™ Particles were then enriched with Dynabeads® MyOne™ Streptavidin C1 Beads (Life Technologies) using an Ion OneTouch™ ES system (Life Technologies). Purified Ion Sphere particles were loaded on an Ion 314 or 318 Chip. Massively parallel sequencing was carried out on a Personal Genome Machine (PGM) sequencer (Ion Torrent™) using the Ion PGM Sequencing 200 Kit version 2 according to the manufacturer's instructions. Sequencing was performed using 500 flow runs that generated approximately 200 bp reads.

### Data analysis

The sequence data were processed using standard Ion Torrent Suite™ Software running on the Torrent Server (Life Technologies, Tokyo, Japan). Raw signal data were analyzed using Torrent Suite™ version 3.6.2 or 4.0.2. The pipeline included signaling processing, base calling, quality score assignment, adapter trimming, PCR duplicate removal, read alignment to human genome 19 reference (hg19), quality control of mapping quality, coverage analysis, and variant calling. Coverage analysis and variant calling used Torrent Variant Caller plugin software (version 3.6 or 4.0) in the Torrent Server. The variant caller parameter setting was germline PGM high stringency. Following data analysis, annotation of single-nucleotide variants, insertions, deletions, and splice site alterations was performed by the Ion Reporter™ Server System (Life Technologies), which identified nonsynonymous mutations. Splice site alteration were analyzed 2 bp upstream or downstream of exon–intron boundaries. Sequence data were visually confirmed with the Integrative Genomics Viewer (IGV) and any sequence, alignment, or variant call error artifacts were discarded. Nonsynonymous mutations were annotated using the BIC database (https://research.nhgri.nih.gov/projects/bic/index.shtml) and ClinVar (http://www.ncbi.nlm.nih.gov/clinvar/) (Landrum et al. 2014). Minor allele frequency was determined from the 1000 Genomes Project database (Abecasis et al. 2012), the 5000 Exome project (http://evs.gs.washington.edu/EVS/), and The Human Genetic Variation Database (HGVD) (http://www.genome.med.kyoto-u.ac.jp/SnpDB).

### Sanger sequencing

PCR was performed using genomic DNA as a template and primer pairs flanking the deleterious variant sites. PCR products were purified using the QIAquick PCR Purification Kit (QIAGEN) according to the manufacturer's instructions. Sequencing was performed with BigDye® Terminator v3.1 using M13 forward or reverse primers (Life Technologies). PCR products were purified and subsequently analyzed by the 3500 Genetic Analyzer (Applied Biosystems, Tokyo, Japan). GenBank sequences of human *BRCA1* (accession number: NP_009225.1) and *BRCA2* (accession number: NP_000050.2) were referred to at the NCBI Reference Sequence Database. Primer sequences are provided in the Table S1.

### Validation NGS data

To assess the consistency of results between laboratories, we sent an aliquot of 25 genomic DNA samples to FALCO Biosystems Ltd. (Kyoto, Japan), and direct sequencing was performed. All variants detected by direct sequencing were interpreted according to the Myriad Genetics’ criteria.

## Results

### Mutation detection of BRCA1 and BRCA2

To analyze the prevalence of HBOC with *BRCA1* and *BRCA2* germline mutations, we recruited 135 breast and/or ovarian cancer survivors and extracted DNA from their peripheral blood lymphocytes. We amplified coding regions and exon–intron boundaries of *BRCA1* and *BRCA2* using multiplex PCR. A total of 167 primer pairs were used, covering 16.25 kb of target genomic sequence. Target sequencing was performed using the Ion Torrent™ PGM System to generate sequence reads of approximately 200 bp. The mean sequencing depth was 495× and the average uniformity of coverage was 98%, which is a sufficient number of reads mapped onto target regions (Table S2).

**Figure 1:**
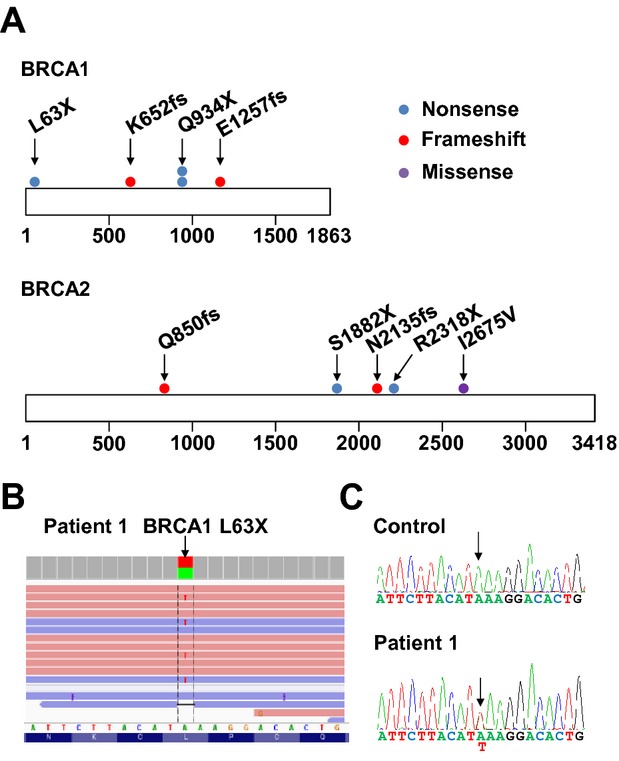
Germline loss of function mutations identified in patients with breast and/or ovarian cancer. (A) Distribution of *BRCA1* and *BRCA2* mutations detected in 10 of 132 patients with breast and/or ovarian cancer. The small circles indicate the number of individuals with a mutation. The number under the square indicates the amino acid position. (B) Representative image of read alignments visualized with IGV. Arrow indicates the germline mutation (*BRCA1* p.L63X, c.188T>A) detected by NGS analysis. (C) Representative validation of NGS data in (B) by Sanger sequencing. Germline mutation (*BRCA1* p.L63X) was detected in the patient but not in the control subject. Arrow indicates position of the mutated nucleotide (c.188T>A).

We used the Torrent Variant Caller plugin to identify 10 truncation variants including nonsense, frameshift insertion/deletion (indel), and splicing alteration mutations (Table1, Fig.1A, B and Fig. S1). To validate the NGS data, we carried out Sanger sequencing of nine probands harboring truncation mutations (Fig.1C and Fig. S2). The mutations identified by NGS were also detected in the nine probands, but not control subjects, by Sanger sequencing (representative individual shown in Fig.1C). This indicated that the NGS analysis used in this study is sufficiently sensitive to detect *BRCA1* and *BRCA2* germline mutations.

**Table 1:** Deleterious mutations found in breast and/or ovarian cancer patients (*n *= 135).

Patient no.	Gene	Designation	Type	Coding	Var Freq (%)	Cov	Ref Cov	Var Cov	BIC	ClinVar
1	*BRCA1*	p.L63X	SNV	c.188T>A	52	564	273	291	CI	Pathogenic
2	*BRCA1*	p.K652fs	INS	c.1952_1953insG	45	448	245	203	–	–
3	*BRCA1*	p.Q934X	SNV	c.2800C>T	53	400	189	211	CI	Pathogenic
4	*BRCA1*	p.Q934X	SNV	c.2800C>T	50	483	241	242	CI	Pathogenic
5	*BRCA1*	p.E1257fs	DEL	c.3770_3771delAG	48	446	231	215	CI	Pathogenic
6	*BRCA2*	p.Q850fs	INS	c.2547_2548insCC	50	161	81	80	–	–
7	*BRCA2*	p.S1882X	SNV	c.5645C>A	53	164	77	87	CI	Pathogenic
8	*BRCA2*	p.N2135fs	DEL	c.6402_6406delTAACT	42	296	173	123	CI	Pathogenic
9	*BRCA2*	p.R2318X	SNV	c.6952C>T	32	91	62	29	CI	Pathogenic
10	*BRCA2*	p.I2675V	SNV	c.8023A>G	56	173	76	97	–	Likely pathogenic

fs, frameshift, SNV, single-nucleotide variant; INS, insertion; DEL, deletion; Var Freq, variant frequency; Cov, coverage; Ref, reference; BIC, Breast Cancer Information Core; CI, clinically important.

### Pathogenic variants in *BRCA1* and *BRCA2* genes

Of the 135 patients enrolled in our study, deleterious *BRCA1* or *BRCA2* truncation mutations were found in 10 (7.4%), including five *BRCA1* mutations (3.7%) and five *BRCA2* mutations (3.7%) (Table1). Of the 10 mutations, five were nonsense, four were frameshift, and one was a missense mutation (Table1). No splice site mutations in the exon–intron boundaries were identified in this study. The *BRCA2* missense mutation p.I2675V (c.8023A>G) was predicted to be causative because it generates a donor site within exon 18 that causes an in-frame deletion and splicing defect (Bonnet et al. 2008). *BRCA1* nonsense mutations p.L63X (c.188T>A) and p.Q934X (c.2800C>T) have previously been recognized as Japanese founder mutations (Sekine et al. 2001; Nakamura et al. 2013). In our study, *BRCA1* L63X and Q934X were found in one and two patients, respectively (Table1), indicating that they occur frequently in the Japanese population. Of the other deleterious mutations identified in this study, one *BRCA1* mutation (p.K652 fs; c.1952_1953insG) and two *BRCA2* mutations (p.Q850 fs; c.2547_2548insCC and p.I2675V; c.8023A>G) were not registered in the BIC database (Table1), suggesting that they are novel. Furthermore, the founder mutations *BRCA1* c.185delAG, *BRCA1* c.5382insC, and *BRCA2* c.6174delT, previously identified in an Ashkenazi Jewish population, were not observed in the current cohort, indicating that *BRCA* mutations vary among different populations as described before (Kim and Choi 2013).

### Missense variants in BRCA1 and BRCA2

NGS analysis detected a total of 23 missense variants of uncertain significance (VUS) including 10 *BRCA1* variants and 13 *BRCA2* variants (Tables2, 3). Of these, six *BRCA1* VUS and eight *BRCA2* VUS are rare variants according to 1000 Genome Project data (<1% population minor allele frequency) (Table2). By contrast, four *BRCA1* and five *BRCA2* variants were present at high frequencies in the study subjects and in ≥1% of the population according to 1000 Genome Project data, indicating that they are common polymorphisms (Table3). We next used HGVD, which contains Japanese exome sequencing data, to examine whether variants identified in this analysis were Japanese genetic variations. One *BRCA1* variant (p.M1628T: 2%) and three *BRCA2* variants (p.K322Q: 1%, p.M784V: 9.7% and p.K2729N: 1.7%) were found in ≥1% of Japanese population (Table2), indicating these variants were comparatively unique to Japanese.

**Table 2:** Rare variants of uncertain significance found in patients with breast and/or ovarian cancer (*n *= 135).

Gene	Designation	Case Freq (*n* = 135)	BIC	Clin Var	dbSNP	1000 genome MAF (%)	5000 exomes	HGVD exome (%)
*BRCA1*	p.L52F	1 (0.73%)	Unknown	Uncertain significance	rs80357084	–	–	0.4
*BRCA1*	p.V271M	1 (0.73%)	Unknown	Uncertain significance	rs80357244	<0.1	–	0.5
*BRCA1*	p.N1018S	1 (0.73%)	–	–	–	–	–	–
*BRCA1*	p.N1236S	1 (0.73%)	–	–	–	–	–	–
*BRCA1*	p.M1628T	6 (4.41%)	Unknown	Conflicting data	rs4986854	0.4	AMAF = 0.02% EMAF = 0.03% GMAF = 0.03%	2
*BRCA1*	p.V1653L	2 (1.47%)	–	–	rs80357261	–	–	–
*BRCA2*	p.K322Q	4 (2.94%)	Unknown	Conflicting data	rs11571640	<0.1	–	1
*BRCA2*	p.M784V	21 (15.4%)	Unknown	Uncertain significance	rs11571653	0.7	–	9.7
*BRCA2*	p.I1929V	1 (0.73%)	NCS	Benign	rs79538375	0.1	AMAF = 0% EMAF = 0.01% GMAF = 0.01%	0.7%
*BRCA2*	p.D1990A	1 (0.73%)	–	Uncertain significance	rs148618542	<0.1	–	–
*BRCA2*	p.G2044V	5 (4%)	Unknown	Conflicting data	rs56191579	0.1	AMAF = 0.02% EMAF= 0% GMAF = 0.01%	–
*BRCA2*	p.V2109I	2 (1.47%)	Unknown	Uncertain significance	rs79456940	<0.1	–	0.7
*BRCA2*	p.V2503I	1 (0.73%)	–	–	–	–	–	0.1^1^
*BRCA2*	p.K2729N	6 (4.41%)	Unknown	Uncertain significance	rs80359065	0.3	–	1.7

MAF, minor allele frequency; AMAF, African American minor allele frequency; EMAF, European American minor allele frequency; GMAF, global minor allele frequency; HGVD, the human genetic variation database.

Allele frequency data from Yokohama City University.

**Table 3:** Common variants found in patients with breast and/or ovarian cancer (*n *= 135).

Gene	Designation	Case Freq (*n* = 135)	BIC	ClinVar	dbSNP	1000 genome MAF (%)	5000 exomes	HGVD exome (%)
*BRCA1*	p.P871L	78 (57%)	NCS	Benign	rs799917	48.3	AMAF = 20% EMAF = 33.59% GMAF = 49.32%	33.4
*BRCA1*	p.E1038G	78 (57%)	NCS	Benign	rs16941	30.3	AMAF = 18.84% EMAF = 32.55% GMAF = 27.90%	33.2
*BRCA1*	p.K1183R	78 (57%)	NCS	Benign	rs16942	32.4	AMAF = 23.83% EMAF = 32.44% GMAF = 29.52%	33.3
*BRCA1*	p.S1613G	78 (57%)	NCS	Benign	rs1799966	32.7	AMAF = 24.26% EMAF = 32.66% GMAF = 29.82%	33.4
*BRCA2*	p.N289H	31 (23%)	NCS	Benign	rs766173	5.8	AMAF = 2.02% EMAF = 3.68% GMAF = 3.12%	13.7
*BRCA2*	p.N372H	46 (34%)	NCS	Conflicting data	rs144848	24.0	AMAF = 12.89% EMAF = 28.59% GMAF = 23.32%	22.3
*BRCA2*	p.N991D	30 (22%)	Unknown	Benign	rs1799944	6.2	AMAF = 3.86% EMAF = 3.66% GMAF = 3.73%	13.5
*BRCA2*	p.V2466A	135 (100%)	Unknown	Uncertain Significance	rs169547	2.2	AMAF = 6.47% EMAF = 0.06% GMAF = 2.23%	99.9
*BRCA2*	p.I3412V	3 (2.2%)	Unknown	Conflicting data	rs1801426	4.3	AMAF = 10.64% EMAF = 0.19% GMAF = 3.73%	2

NCS, not clinically significance; MAF, minor allele frequency; AMAF, African American minor allele frequency; EMAF: European American minor allele frequency; GMAF, global minor allele frequency; HGVD, the human genetic variation database.

### Cross-validation of NGS data

To further analysis the accuracy of NGS data, we outsourced a set of 25 samples for genetic analysis and compared the results at the two independent laboratories (our laboratory and FALCO Biosystems Ltd.). The result performed with two institutions showed concordance was 100% for all 25 samples (Table4), therefore, we confirmed the reproducibility of our NGS data. Functional significance of five nonsense mutation, four indels and sixteen missense variants were interpreted by FALCO Biosystems (the Myriad Genetics’ criteria). According to this, all nonsense mutation and indels were interpreted as deleterious and one missense variant is suspected deleterious (Table4). In contrast, five missense variants were uncertain one was favor polymorphism and nine were polymorphism (Table4). Interestingly, five missense variants (BRCA1: p.L52F, p.N1018S, p.N1236S, p.V1653L, BRCA2: p.D1990A) interpreted as an uncertain were rare variants according to HGVD database (Table2). Although it remains unclear whether these five missense VUS have influence on BRCA1 or BRCA2 functions, it is possible that some of variants are associated with HBOC.

**Table 4:** Comparison data from two independent laboratories.

	NGS data from our laboratory		FALCO biosystems		
Gene	Designation	Coding	Designation	Coding	Interpretation
*BRCA1*	p.L63X	c.188T>A	p.L63X	c.188T>A	Deleterious
*BRCA1*	p.K652fs	c.1952_1953insG	p.K652fs	c.1952_1953insG	Deleterious
*BRCA1*	p.Q934X	c.2800C>T	p.Q934X	c.2800C>T	Deleterious
*BRCA1*	p.Q934X	c.2800C>T	p.Q934X	c.2800C>T	Deleterious
*BRCA1*	p.E1257fs	c.3770_3771delAG	p.E1257fs	c.3770_3771del	Deleterious
*BRCA2*	p.Q850fs	c.2547_2548insCC	p.Q850fs	c.2547_2548insCC	Deleterious
*BRCA2*	p.S1882X	c.5645C>A	p.S1882X	c.5645C>A	Deleterious
*BRCA2*	p.N2135fs	c.6402_6406delTAACT	p.N2135fs	c.6402_6406delTAACT	Deleterious
*BRCA2*	p.R2318X	c.6952C>T	p.R2318X	c.6952C>T	Deleterious
*BRCA2*	p.I2675V	c.8023A>G	p.I2675V	c.8023A>G	Suspected deleterious
*BRCA1*	p.L52F	c.154C>T	p.L52F	c.154C>T	Uncertain
*BRCA1*	p.V271M	c.811G>A	p.V271M	c.811G>A	Polymorphism
*BRCA1*	p.N1018S	c.3053A>G	p.N1018S	c.3053A>G	Uncertain
*BRCA1*	p.N1236S	c.3707A>G	p.N1236S	c.3707A>G	Uncertain
*BRCA1*	p.M1628T	c.4883T>C	p.M1628T	c.4883T>C	Polymorphism
*BRCA1*	p.V1653L	c.4957G>T	p.V1653L	c.4957G>T	Uncertain
*BRCA2*	p.K322Q	c.964A>C	p.K322Q	c.964A>C	Polymorphism
*BRCA2*	p.M784V	c.2350A>G	p.M784V	c.2350A>G	Polymorphism
*BRCA2*	p.I1929V	c.5785A>G	p.I1929V	c.5785A>G	Polymorphism
*BRCA2*	p.D1990A	c.5969A>C	p.D1990A	c.5969A>C	Uncertain
*BRCA2*	p.G2044V	c.6131G>T	p.G2044V	c.6131G>T	Polymorphism
*BRCA2*	p.V2109I	c.6325G>A	p.V2109I	c.6325G>A	Polymorphism
*BRCA2*	p.V2503I	c.7507G>A	p.V2503I	c.7507G>A	Favor Polymorphism
*BRCA2*	p.K2729N	c.8187G>T	p.K2729N	c.8187G>T	Polymorphism
*BRCA2*	p.I3412V	c.10234A>G	p.I3412V	c.10234A>G	Polymorphism

### *BRCA2* germline mutations in unaffected individuals

**Figure 2:**
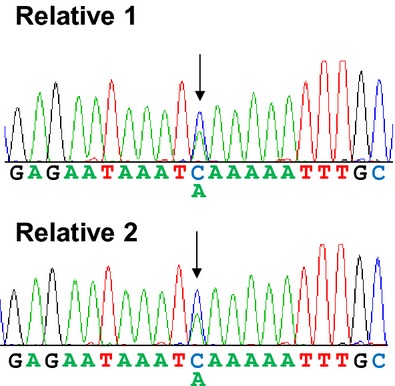
Pedigree of subjects with germline *BRCA2* mutations. PCR followed by Sanger sequencing were performed on genomic DNA from two unaffected relatives. The nonsense mutation (*BRCA2* S1882X) found in the proband was also detected in both relatives. Arrows indicate the position of the missense mutation in the relatives.

To determine whether unaffected relatives carry the same deleterious mutations as the probands, we enrolled two unaffected relatives of an ovarian patient harboring *BRCA2* p.S1882X (c.5645C>A). Sanger sequencing analysis showed that both relatives also carried the *BRCA2* mutation (Fig.2). Taken together, these results suggested the screening of BRCA1/2 mutation carrier using NGS has a role in aiding cancer prevention and reducing the cancer risk in unaffected individuals.

## Discussion

In this study, we determined the sequence of *BRCA1* and *BRCA2* in 135 breast and/or ovarian cancer patients by NGS, which was then validated using Sanger sequencing. We identified deleterious *BRCA1* or *BRCA2* truncation mutations in 10 patients, including one *BRCA1* (p.K652 fs; c.1952_1953insG) and two *BRCA2* (p.Q850 fs; c.2547_2548insCC and p.I2675V; c.8023A>G) mutations, which, to the best of our knowledge, are novel. An aliquot of 25 samples were outsourced for genetic analysis and there was concordance in results obtained by two independent laboratories. Furthermore, two unaffected relatives were shown to carry the same deleterious *BRCA2* mutation observed in the proband.

This study demonstrated the utility of NGS for performing the molecular diagnosis of HBOC based on *BRCA1* and *BRCA2* genetic alterations. We propose that the technique is suitable to detect mutations in tumor suppressor genes such as *BRCA1* and *BRCA2* where causative mutations are distributed throughout the genes (Costa et al. 2013). It is also likely to be informative in the analysis of the many other susceptible genes related to breast and ovarian cancer that have been discovered (Couch et al. 2014). Indeed, several studies have developed a system for the simultaneous detection of multiple target genes using NGS (Walsh et al. 2010, 2011; Castéra et al. 2014). The identification of mutations in genes other than *BRCA1* and *BRCA2* will also help understand the genetic heterogeneity and penetrance of HBOC (Walsh et al. 2010; Castéra et al. 2014).

Identifying founder mutations would enable us to examine specific loci in the screening of high-risk subpopulations for inherited breast and ovarian cancer without performing a full sequence analysis of *BRCA1* and *BRCA2*. Founder mutations have previously been described in an Ashkenazi Jewish population in which 3% of individuals carried *BRCA1* c.185delAG, *BRCA1* c.5382insC, or *BRCA2* c.6174delT mutations (Ferla et al. 2007). The KOHBRA study carried out large population in Korea showed BRCA2 p.R2494X, BRCA1 p.Y130X, and BRCA1 p.V1833 fs were candidate founder mutations (Han et al. 2011). In the Japanese population, *BRCA1* L63X and Q934X were reported as founder mutations (Ikeda et al. 2001; Sekine et al. 2001; Sugano et al. 2008; Nakamura et al. 2013), and were observed in three patients of the present study. Although another study showed that BRCA2 5802delTTAA mutation was considered as common in Japanese breast cancer patients (Ikeda et al. 2001), we did not detect this type of mutation. These observations implies that the *BRCA1* and *BRCA2* founder mutation status in the Japanese population differs from that of other countries. Because *BRCA1* and *BRCA2* genetic testing is less commonly undertaken in Japan compared with western countries, a large-scale cohort study is required to obtain more precise information about founder mutations in Japan.

This study demonstrated, among 135 breast and/or ovarian cancer patients, 10 patients (7.4%) were deleterious carriers. Previous study with Japanese subject showed BRCA1 and BRCA2 germline mutation in 36 subjects out of 135 (26.8%) (Sugano et al. 2008). In our study, the proportion of mutation carriers was lower than that of previous study, because we enrolled the subjects at diagnosis and not selected for family history.

Large genomic alterations in *BRCA1* and *BRCA2* are pathogenic, but a limitation of our study was that we did not examine these gene rearrangements. Therefore, additional analysis such as multiplex ligation-dependent probe amplification is required. In western countries, large genomic rearrangements were frequently found in *BRCA1* gene locus (Gad et al. 2002; Montagna et al. 2003). Contrary to these findings, large genomic deletion were thought to be rare in Japanese population (Sugano et al. 2008). We expect that the combinatorial use of the NGS system with large genomic analysis will be desirable for screening HBOC.

In conclusion, we showed that multiplex PCR followed by NGS is useful for screening *BRCA1* and *BRCA2* germline mutations of probands and could be applicable to cancer prevention in unaffected relatives carrying the same mutation. This method is both cost- and time-effective for the screening of genetic variants, and will be beneficial in clinical and diagnostic use.

## Supporting Information

**Figure S1.** Deleterious mutation identified by NGS data. Image of read alignments visualized with IGV. Arrows indicate the position of the mutations in the patients.

**Figure S2.**DNA sequencing analysis of deleterious *BRCA1* and *BRCA2* mutations. PCR followed by Sanger sequencing validated the nonsense mutations and frameshift insertions/deletions using genomic DNA from control subjects and each patient. Arrows indicate the position of the nonsense mutation in the patient and the corresponding position in the control subject.

**Table S1.** Primer sequences used for Sanger sequencing.

**Table S2.** Coverage analysis data.
