# Supplementary figures and images for: Detection of BRCA1 and BRCA2 germline mutations in Japanese population using next-generation sequencing
Source: Mol Genet Genomic Med. 2014 Dec 4;3(2):121–9. doi: 10.1002/mgg3.120 (PMC4367084; doi:10.1002/mgg3.120)

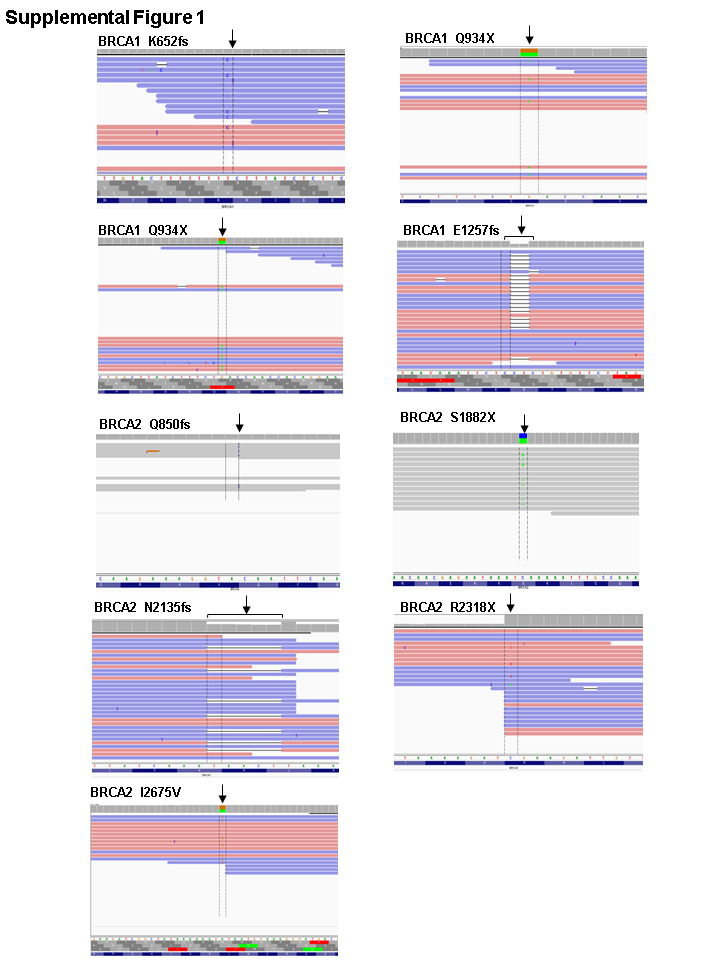

Supplement: Supplementary file 1 [file mgg30003-0121-sd1.tif]

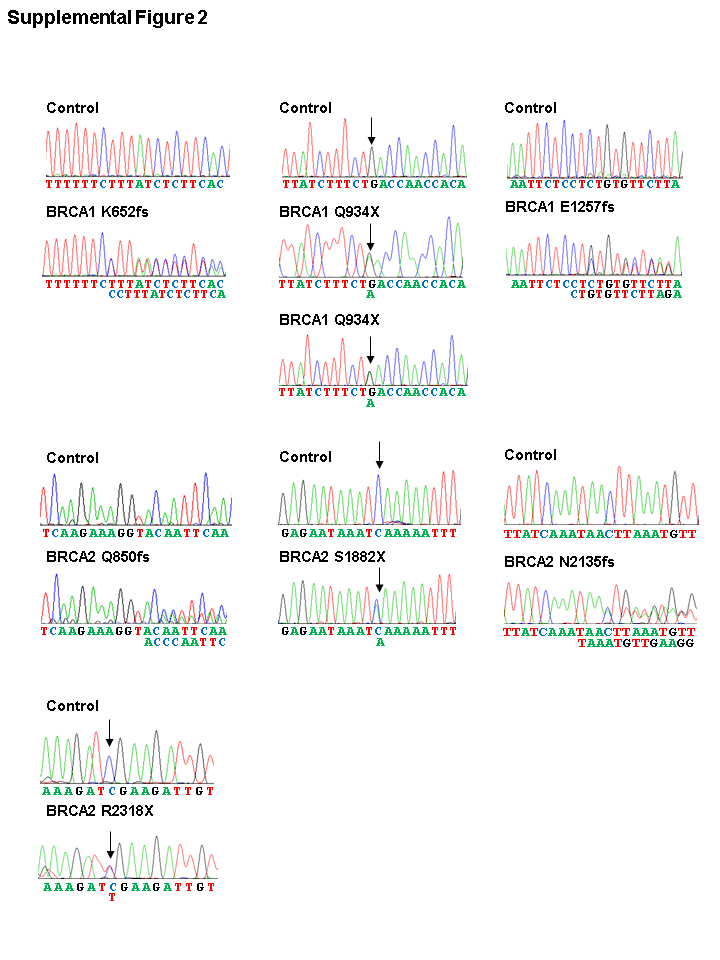

Supplement: Supplementary file 2 [file mgg30003-0121-sd2.tif]
